# Characteristics and background mucosa status of early gastric cancer after *Helicobacter pylori* eradication: A narrative review

**DOI:** 10.1097/MD.0000000000031968

**Published:** 2022-12-02

**Authors:** Yali Wei, Chen Jiang, Yiping Han, Wen Song, Xiaoyu Li, Xiaoyan Yin

**Affiliations:** a Department of Gastroenterology, the Affiliated Hospital of Qingdao University, Qingdao, Shandong Province, China.

**Keywords:** background mucosa status, early gastric cancer, *Helicobacter pylori* eradication

## Abstract

Helicobacter pylori (*H pylori*) eradication treatment can reduce the risk of gastric cancer. However, early gastric cancer (EGC) can still be detected after eradication. Meanwhile, EGC after eradication is challenging to diagnose by an endoscopist in some cases due to the lack of apparent characteristics and the complex mucosal status.

This review aims to summarize the endoscopic and histological characteristics and the mucosal risk factors for gastric cancer after *H pylori* eradication.

The literature was searched for possible reported gastric cancer after eradication in “PubMed.” These included related clinical studies and reviews, and unrelated or non-English articles were excluded.

Endoscopically, EGC displays a small, reddish and depressed lesion, indistinct border, “gastritis-like” appearance and submucosal invasion. Histologically, it is divided into surface differentiation, nontumorous epithelium, and intestinal type. The risk factors include severe gastric atrophy, intestinal metaplasia in the corpus, and map-like redness.

In conclusion, these studies on the characteristics and risk mucosal factors of patients with gastric cancer after *H pylori* eradication will drive the establishment of a novel endoscopic surveillance and diagnosis system for *H pylori*-eradicated patients.

## 1. Introduction

Currently, gastric cancer remains the fifth most common and the fourth most common cause of mortality worldwide, with an especially high incidence in Eastern Asia.^[1]^ In 1983, Robin Warren and Barry Marshal^[2]^ first discovered Helicobacter pylori (*H pylori*) and proposed that it was related to gastritis-associated diseases, including peptic ulcer and gastric cancer. In 1994, *H. pylori* was categorized as a definite gastric carcinogen.^[3]^ Therefore, gastric cancer is an inflammation-associated carcinoma promoted by *H pylori* infection, characterized by ongoing chronic gastritis, development of gastric atrophy (GA), formation of intestinal metaplasia (IM), and finally, dysplasia and cancer.^[4–6]^ Then, a relative consensus advocated that *H pylori* eradication could improve mucosal inflammation and reduce the incidence rates of gastric cancer.^[6–11]^ Thus, on reports, *H pylori* eradication has shifted from treatment to primary prevention of gastric cancer on reports.^[10–13]^

However, the risk of progression to gastric cancer still exists after successful *H pylori* eradication. Gastric cancer after *H pylori* eradication is defined as early gastric cancer detected after more than 1 year of successful *H pylori* eradication, which contains primary gastric cancer and metachronous gastric cancer.^[14]^ There are no statistical studies on the incidence of gastric cancer after eradication based on a large population, but other studies could calculate the incidence. Gastric cancer after *H pylori* eradication occurred at a rate of 1.1% (20 of 1787) during a 9-years prospective follow-up study in Japan.^[15]^ Choi et al^[16]^ reported that metachronous gastric cancer was reduced by *H pylori* eradication in an open-label, prospective, randomized controlled trial. However, 18 gastric cancer cases were detected in 437 patients in the eradication group (4.1%). Another prospective, double-blind, placebo-controlled, randomized trial found that the incidence of gastric cancer reached 7.2% (14 of 194) in the treatment group.^[17]^

The study of gastric cancer after *H pylori* eradication has received great attention. Related reports have also elucidated the critical fact that there is a significant difference in characteristics between Hp-eradication and Hp-positive gastric cancer. In terms of diagnosis, an endoscopic examination could be completed, during which the characteristics and biopsy could be assessed to define the histological diagnosis.^[18]^ In terms of treatment, endoscopic resection could be considered a definitive treatment for most early gastric cancer patients in Asia.^[19,20]^ However, the special endoscopic and pathological manifestations in early gastric cancer (EGC) after eradication may obscure the tumor discovery and diagnosis for an endoscopist.

Based on the above findings, gastric cancer after eradication has become a new challenge in the clinic. This narrative review aims to summarize the characteristics of gastric cancer after *H pylori* eradication and the mucosal risk factors. The findings of this review will be beneficial to establish a systematic endoscopic surveillance and diagnosis system in the clinic for *H pylori* eradicated patients.

## 2. Methods

A literature search was performed using “PubMed,” The main keywords for the literature search were “Gastric Cancer,” “Helicobacter pylori Eradication,” Endoscopic,” Histological,” “Risk Factor,” “Predictor,” Gastric Mucosal,” “Gastric atrophy,” “Intestinal Metaplasia,” “Mottled Patchy Erythema,” and “Map-like Redness.” The author attempted to collect data from manuscripts mainly published in the past 10 years so that the most recent information could be incorporated into this manuscript. Nevertheless, 2 manuscripts published beyond 10 years were also included, as they contained information about gastric cancer after eradication. As this is a narrative review, ethical approval was not needed.

## 3. Results

### 3.1. Endoscopic characteristics

The characteristics of gastric cancer after *H pylori* eradication differ from those of lesions with infection. The middle and lower locations, smaller and reddish lesions, and morphology tended to be depressed, with an indistinct border, “gastritis-like” microstructure, microvascular appearance, and submucosal invasion found on endoscopy.

#### 3.1.1. *Location*.

There is still no clear location distribution for ECG findings after *H pylori* eradication. A 9-years follow-up study only found 20 cases, of which 16 were noncardiac cancers (8 in the antrum, 5 in the angulus, and 3 in the corpus) and 4 were cardiac cancers.^[15]^ Meanwhile, a population-based cohort study confirmed that 95 noncardiac cancers accounted for 62.1% after eradication.^[21]^ Another study found that 80% of lesions were present in the middle and lower anatomical locations.^[22]^ However, the prevalence of cases in the upper anatomical position in the eradication group was higher than that in the control group (21.6% and 10.7%, respectively). It may be relevant that *H pylori* was inversely associated with the risk of cardia cancer.^[23]^ However, in another multicenter and matched study, there was no statistical significance in location.^[14]^ However, these lesions were mainly distributed in the middle and lower areas.

#### 3.1.2. *Size*.

The inhibition trend of EGC after *H pylori* eradication is shown in terms of tumor size. Keiko et al^[24]^ reported a small average diameter in the eradication group (mean = 9.5 mm). Kazutoshi et al^[25]^ also confirmed that the size of the eradication group was smaller than that of the infection group and that tumors of 11 mm or less were more frequent in the ROC analysis. Other studies further validated this result and found that the mean tumor size was approximately 11 mm in the EGC group after eradication.^[22,26]^ However, Maehata et al^[14]^ showed that the tumor size of metachronous cancers in the eradication group was smaller (mean = 11 mm) than that of primary cancers (mean = 14 mm). Differences in study design and data sources for each study may have led to the reported difference in tumor size. However, it could be confirmed that the gastric cancer after eradication was small, which may be associated with *H pylori* eradication inhibiting tumor growth.^[24,26,27]^

#### 3.1.3. *Tumor color*.

Kazutoshi et al^[25]^ divided the tumor color into whitish, reddish, and intermediary. The proportion of whitish color in the eradication group was smaller than that in the infected group. It was also proposed that no whitish color was an independent feature of EGC. Another study directly proposed that the reddish appearance on endoscopy is one of the clinical features of EGC after *H pylori* eradication.^[22]^

#### 3.1.4. *Morphology*.

Flat or depressed morphology can be found in EGC after *H pylori* eradication. I to et al^[28]^ studied the morphological changes in tumors before and after eradication and found that the elevated lesions became flat, but the depressed cases were not. This change was confirmed in subsequent long-term prospective and multicenter studies.^[14,29]^ Kazutoshi et al^[25]^ compared the eradication and infection groups and found a flattening trend. In 2016, a study divided the morphology into protruded (0–I, 0–IIa) and depressed (0–IIc, 0–IIa + IIc, and 0–IIc + IIa).^[22]^ Later, combined with the Paris classification, morphology was classified as the elevated type, including 0 to I, IIa, and IIa + IIc, and the depressed type, including 0 to IIb, IIc, IIc + IIa, IIc + III, and III.^[14]^ Together, these results confirmed that the eradication group tended to be depressed. Therefore, flat, or depressed morphology, which may be related to *H pylori* eradication inhibits upward (expansive) growth.^[28]^

#### 3.1.5. *Microstructure (MS), microvascular (MV), and border*.

**Figure 1.**
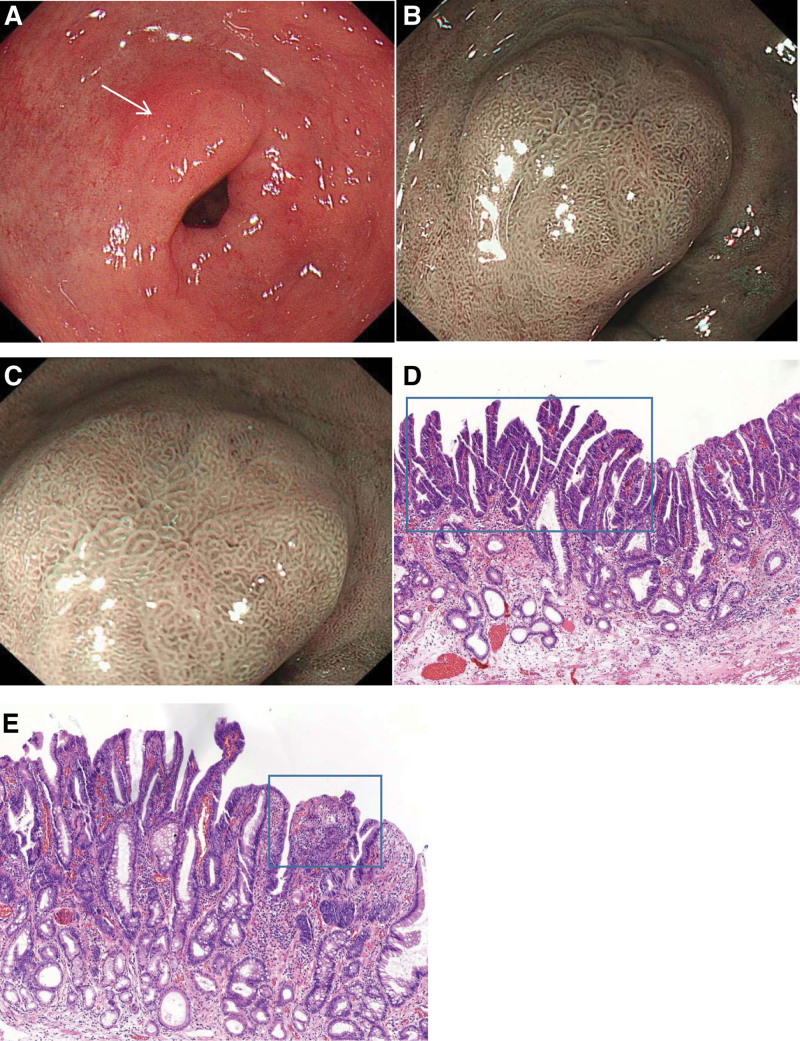
These findings were obtained from a patient after successful eradication. (A) WLE findings: A reddish and slightly excavated lesion was found on the small, curved side of the antrum (white arrow). (B and C) NBI-ME findings: The lesion presented gastritis-like appearance, with regular MS, MV, and clear demarcation. (D) Histopathological findings: The glands in the blue square area showed a villous structure that can be diagnosed as low-grade neoplasia, and the remaining glands have a nontumorous structure. (E) Histopathological findings: Many disordered, differently sized, darker nuclei can be observed in the blue square area, with visible nucleoli, which can be identified as high-grade neoplasia. MS = microstructure, MV = microvascular, NBI-ME = the narrow-band imaging with magnifying endoscopy, WLE = white light endoscopy.

The narrow-band imaging with magnifying endoscopy (NBI-ME) clearly shows the MS and MV. Masaaki et al^[30]^ proposed a definition of “gastritis-like” appearance under NBI-ME as MS mixed papillae and pits with a regular (open/closed-loop) or faint MV, similar to the surrounding noncancerous mucosa (Fig. 1). The study also found that the eradication group (n = 22,44.0%) appeared more frequently than the control group (n = 2,4.0%). In addition, another study suggested that the “gastritis-like” appearance was mainly distributed in the area of gastric cancer in the eradication group, which also contains the edge of the lesion and the indistinct border, making it difficult to distinguish from the surrounding mucosa.^[31]^

#### 3.1.6. *Invasive depth*.

Although size and morphology were inhibited after *H pylori* eradication, the invasive depth was aggressive. Kazutoshi et al^[25]^ found that the tumors were deeper and showed more frequent submucosal invasion in the eradication group. A multicenter propensity score-matched study also found a higher trend of submucosal invasion with eradication than with infection.^[14]^ Although significant differences were not observed in the 2 groups described by the Japanese study, the lesions of predominant intestinal type in this eradication group were submucosal invasion rate tended to be higher.^[24]^

### 3.2. *Histological characteristics*

The review revealed that the histological characteristics of gastric cancer after *H pylori* eradication were also typical, including surface differentiation, nontumorous epithelium, and intestinal type.

#### 3.2.1. *Surface differentiation*.

The surface differentiation indicated that Ki-67 positive cells were restricted to the middle or lower layer in a cancer. The absence of Ki-67-positive cells in the surface layer of the tumor promotes maturation at the surface.^[30]^ The “gastritis-like” appearance could be related to surface differentiation.^[30,31]^ Keiko et al^[24]^ found that Ki-67-positive cells were lower in the eradication group than in the control group. It was speculated that the surface of the tumor tended to mature after eradication, related to the inhibition of proliferation growth.^[32]^

#### 3.2.2. *Nontumorous epithelium (NE): epithelium with low-grade atypia (ELA*).

The normal columnar epithelium appearing on the tumor surface. Namely, NE or ELA, was first discovered in 2005.^[28]^ Then, Ki-67 markers were not found by immunohistochemical staining in ELA, which indicated maturation in the epithelium of gastric cancer after successful eradication.^[32]^ Saka et al^[31]^ further divided the proportion of ELA in cancerous areas into different grades and found that level 0 (≤ 10%) occurred more frequently in the control group. Still, levels 1 (50% – 10%) and 2 (>50%) were significantly higher in the eradication group. Therefore, the eradication group not only found the ELA but also occupied an extensive range in the cancerous areas. Next, according to the location and structure, NE was classified into full gland, marginal surface and internal surface types. Noda et al^[33]^ found that only internal surface type was significantly linked to *H pylori*-eradicated cancer.

#### 3.2.3. *Intestinal type*.

The Lauren classification system includes intestinal and diffuse types. A 9-years prospective follow-up study in Japan reported 15 intestinal types in 20 patients after *H pylori* eradication.^[15]^ Another retrospective study reported 20 cases of intestinal gastric cancer (95%) and 1 diffuse type (5%).^[34]^ The cause may be that neutrophil infiltration was depressed after *H pylori* eradication, which was a risk factor for diffuse gastric cancer.^[35]^

In summary, these characteristics of EGC after *H pylori* eradication, including the small lesion, flat or depressed morphology, and “gastritis-like” appearance under endoscopy and the surface differentiation, nontumorous epithelium in histology, maybe because of the proliferative ability of the tumor was inhibited after *H pylori* eradication. This ability can be evaluated using Ki-67, Wnt5, and serum gastrin.^[36,37]^ Some papers have found that the Ki-67 index promoting the proliferation of tumor cells was lower in the eradicated group than in the positive group.^[24,38]^ In addition, Matsuo et al^[27]^ proposed that Wnt5a stimulating invasion of tumor cells was lower in the eradicated group. A recent article speculated that decreased levels of serum gastrin, a growth factor for gastric epithelial cells, may play an essential role in these characteristics, especially in terms of depression and flat morphology.^[39]^

### 3.3. *Background mucosa status*

There are potential benefits after *H pylori* eradication in background mucosa status, including reduced mucosal inflammation, delayed mucosal damage, and improved mucosal function.^[7,12,40–42]^ It showed that diffuse redness, spotty redness, mucosal edema, and enlarged folds were improved under endoscopy.^[43]^

However, whether *H pylori* eradication can improve AG and IM remains unclear. A meta-analysis published in 2011 retrieved relevant papers about morphologic changes, particularly in AG and IM before and after *H pylori* eradication, and proposed an improved GA in the corpus, except for GA in the antrum and IM.^[44]^ Later, published articles explored whether *H pylori* eradication can improve AG and IM.^[45–49]^ For a controversial discussion, these articles suggested that differences AG and IM recovery levels resulted from different follow-up times.^[43,47,50]^ Therefore, some studies have also shown that mucosa status in prolonged follow-up can observe the expected effect, but there is still a risk of developing gastric cancer.^[51–53]^

Another change after *H pylori* eradication is map-like redness or mottled patchy erythema (multiple slightly flat or depressed erythematous lesions).^[54,55]^ For this phenomenon, papers have also speculated that rapidly recovered non-IM/AG areas make the relative red IM/AG areas more visible after successful *H pylori* eradication.^[43,55]^

#### 3.3.1. *Severe gastric atrophy (GA*).

**Figure 2.**
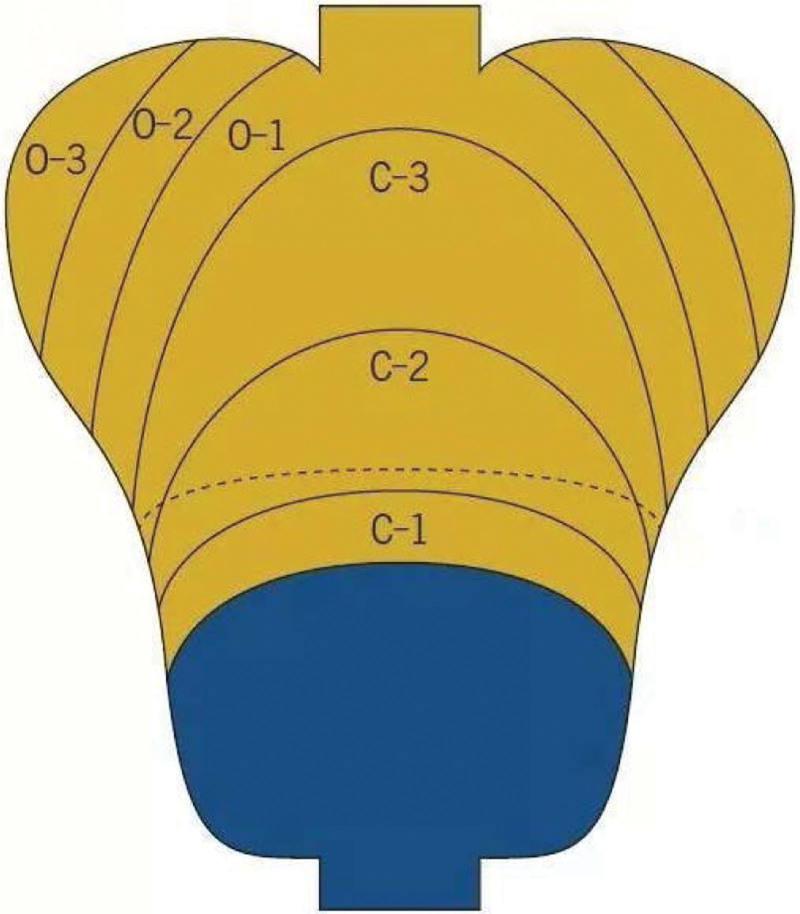
According to the location and extent of gastric atrophy, the Kimura-Takemoto classification was classified as closed (*C*-1, *C*-2, *C*-3) and open (*O*-1, *O*-2, *O*-3).

**Figure 3.**
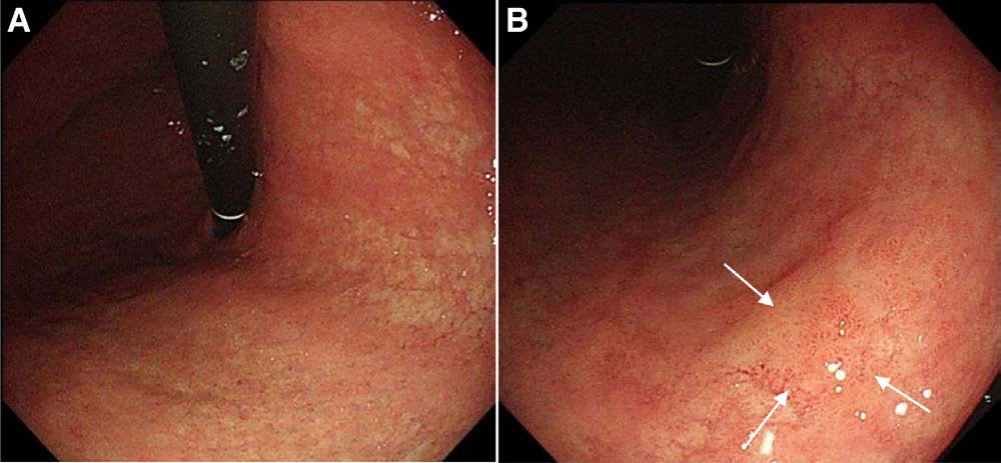
(A) Shown is a case of open-type gastric atrophy (*O*-1) after eradication. (B) In this mucosa status (*O*-1), a suspicious lesion corresponding to the white arrow area with yellow color and slightly depressed type was found on the small curved side of the corpus, and the pathology suggested high-grade neoplasia.

GA is a risk factor for gastric cancer development during *Helicobacter pylori* infection.^[56]^ It is improved by removing risk factors such as *H pylori*, except for severe GA.^[57]^ Satoki et al^[34]^ assessed 21 cases of *H pylori* eradication according to the Kimura-Takemoto classification system^[58]^ (Fig. 2). They found that the incidence of gastric cancer increased as the level of GA increased. Another study confirmed that severe GA (*O*-2, *O*-3) was an independent risk factor for gastric cancer after *H pylori* eradication.^[59]^ The latest study based on the Kimura-Takemoto classification, using the Kyoto classification of gastritis, found that severe GA (A2 score in Kyoto gastritis classification [*O*-1, *O*-2, *O*-3], open-type in Kimura-Takemoto classification) was an essential endoscopic marker (Fig. 3).^[60]^ Therefore, severe GA is a direct risk factor for gastric cancer development after *H pylori* eradication.

#### 3.3.2. *Intestinal metaplasia (IM) of the corpus*.

IM is another meaningful risk factor. Satoki et al^[34]^ divided IM into Group A (no IM), Group B (IM only in the antrum), and Group C (in the corpus with or without antrum). They confirmed that a wider range of IM increased the risk of gastric cancer, especially IM in the corpus.^[61]^

#### 3.3.3. *Map-like redness (Mottled patchy erythema [MPE]*).

**Figure 4.**
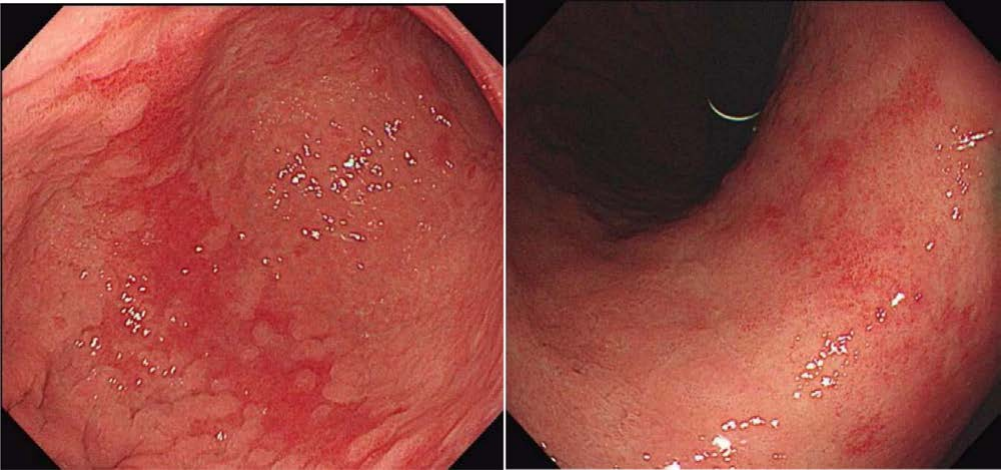
These 2 pictures were map-like redness from the same patient.

MPE is defined as multiple flat or depressed erythematous lesions. Moribata et al^[54]^ found that map-like redness may be a positive predictor of early gastric cancer after *H pylori* eradication (Fig. 4). Majima et al^[62]^ further verified positive risk factors using linked color imaging. In addition, Nagata et al^[55]^ proposed MPE, which was consistent with map-like redness under endoscopy. Therefore, it is a key index for detecting EGC after *H pylori* eradication during endoscopy.

## 4. Discussion

Based on these findings, the characteristics of EGC after *H pylori* eradication, including the small lesion, flat or depressed morphology and “gastritis-like” appearance under endoscopy, showed that the proliferative capability of the tumor might be suppressed by *H pylori* eradication, which increases the difficulty for endoscopists. Hence, doctors should be familiar with the characteristics which is beneficial for the early detection of lesions in *H pylori*-eradicated patients. In addition to understanding the characteristics, applying suitable technology is necessary. As a traditional examination, white light endoscopy (WLE), is sufficient for detecting some features of lesions, including location, size, color, morphology, and border.^[63]^ NBI-ME can visualize the MV and MS structures compared with WLE.^[64]^ The findings demonstrate that NBI-ME has significantly superior diagnostic accuracy to WLE for gastric cancer after *H pylori* eradication, especially for lesions with a “gastritis-like” appearance.^[33,65]^ Furthermore, blue laser imaging (BLI) is a new image-enhanced endoscopy technique that uses laser illumination to change brightness and contrast.^[66,67]^ BLI has improved diagnostic performance for lesions compared with NBI in brightness and contrast.^[68]^ Probe-based confocal laser endomicroscopy, an imaging technique used to obtain real-time optical biopsies, outperformed ME-NBI in diagnosing the horizontal extent of ambiguous lesions.^[69]^ However, BLI and probe-based confocal laser endomicroscopy are not fully promoted in the clinic, which may be restricted in technology. Large-scale clinical experiments are not performed to evaluate the actual effect in detecting lesions. Because *H pylori*-eradicated cancer has different characteristics from *H pylori*-infected cancer, endoscopists should be aware of and choose the appropriate endoscopic examination for different lesions.

Histological characteristics may be partially responsible for the reduced diagnostic reliability of EGC after *H pylori* eradication. NE and surface differentiation are typical histological findings of gastric cancer after *H pylori* eradication,^[70]^ which are linked with a “gastritis-like” appearance. Therefore, tissue biopsy must be performed in *H pylori*-eradicated patients with a “gastritis-like” appearance under endoscopy. Furthermore, a study investigated whether the duration might influence the extent to which NE covers the lesion after eradication and whether gastritis-like appearance is more frequent at the lesion margin.^[31]^ Even if a suspicious lesion is detected under endoscopy, there is interference in delineating the border between the tumorous and the nontumorous mucosa. Therefore, endoscopists should be alert to the extent and border of the “gastritis-like” appearance in patients with longer durations after eradication. The extent of the lesion should be appropriately expanded by using ME-NBI to accurately delineate the border and to achieve treatment effectiveness.

Analysis of background mucosa status found that severe GA, IM in the corpus, and map-like redness could be risk factors for EGC after *H pylori* eradication. Therefore, the above background mucosa status detected in the patients cured after eradication indicated an early warning effect. Although suspicious lesions are not detected in the first endoscopy examination, regular endoscopic monitoring is also an essential measure. The monitoring time from eradication to cancer occurrence is not clearly defined. NishizawaT et al^[26]^ showed that improvement in endoscopic gastritis with *H pylori* eradication might contribute to the detection of gastric cancer within 1 year after eradication. A retrospective study found that EGC should be vigilant after the fifth year following eradication.^[71]^ Take et al^[72]^ found that endoscopic surveillance for gastric cancer after eradication should be continued beyond 10 years. Hence, early and longer endoscopic monitoring may produce the expected clinical effects for *H pylori*-eradicated patients with mucosal risk factors.

**Figure 5.**
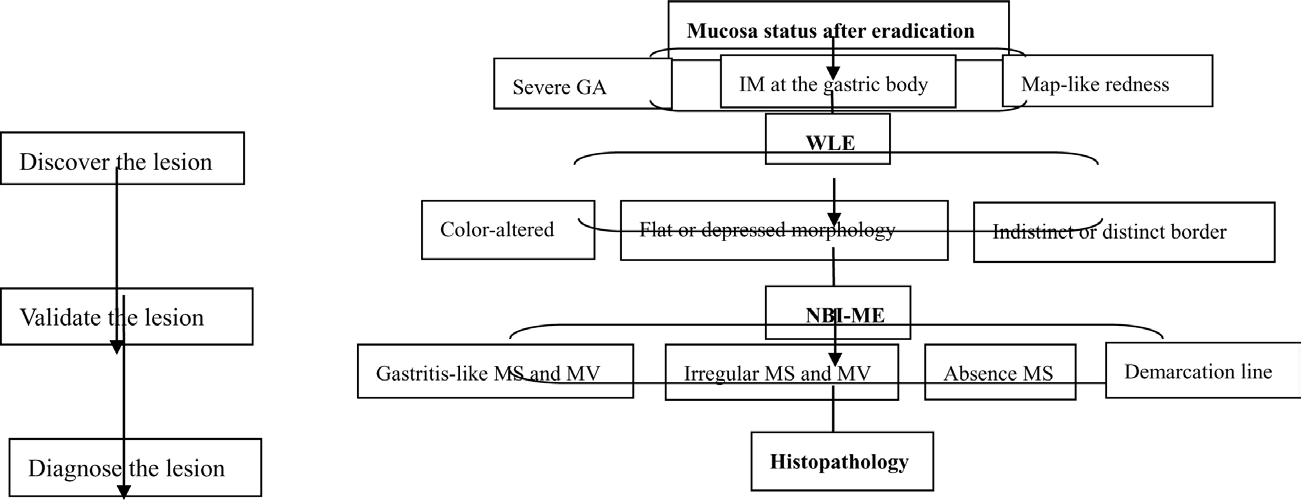
The screening of early gastric cancer after Helicobacter pylori eradication.

In conclusion, severe GA, IM in the corpus, and map-like redness have been proven helpful in predicting gastric cancer after *H pylori* eradication. Based on this background mucosa, the color-altered, flat and depressed lesions should be vigilant in WLE, and suspicious lesions covered by the “gastritis-like” changed MS and MV should not be ignored in ME-NBI, especially for the definition of the border, which can be appropriately expanded (Fig. 5). Meanwhile, annual endoscopic surveillance and extended endoscopic follow-up duration would be desirable for *H pylori*-eradicated patients with mucosal risk factors.

The strength of our review is that the characteristics and risk mucosal factors of gastric cancer after eradication were systematically summarized. Meanwhile, we provide novel endoscopic surveillance and diagnosis strategies for *H pylori*-eradicated patients.

In the next study, it is necessary to continue focusing on the characteristics of EGC and background mucosal status after *H pylori* eradication based on data from many samples. Advanced endoscopic techniques should be promoted in the clinic to achieve early diagnosis and treatment. Furthermore, mechanistic research on the characteristics of gastric cancer after eradication should be explored in the future.

## Author contributions

**Conceptualization:** Xiaoyan Yin.

**Data curation:** Yali Wei.

**Formal analysis:** Yali Wei.

**Investigation:** Chen Jiang.

**Supervision:** Chen Jiang, Xiaoyu Li.

**Validation:** Chen Jiang, Wen Song, Xiaoyu Li, Xiaoyan Yin.

**Visualization:** Chen Jiang, Wen Song, Xiaoyu Li, Xiaoyan Yin.

**Writing – original draft:** Yali Wei, Yiping Han.

**Writing – review & editing:** Yali Wei, Yiping Han.
